# Human–Wildlife Interactions: Cultural Sensitivities and Perspectives Influence the Conservation of the Philippine Brown Deer (*Rusa marianna* Desmarest, 1822)

**DOI:** 10.3390/ani15233397

**Published:** 2025-11-25

**Authors:** Jhonnel P. Villegas, Lenilyn R. Pua, Aneta Vidláková, Francisco Ceacero

**Affiliations:** 1 Department of Animal and Wildlife Sciences, Faculty of Tropical AgriSciences, Czech University of Life Sciences Prague, Kamýcká 129, 165 00 Prague, Czech Republic; villegas@ftz.czu.cz (J.P.V.);; 2 Faculty of Teacher Education, Davao Oriental State University, City of Mati 8200, Davao Oriental, Philippines; 3 Faculty of Advanced and International Studies, Davao Oriental State University, City of Mati 8200, Davao Oriental, Philippines

**Keywords:** Aliwagwag, ancestral domain, Cervidae, Eastern Mindanao, indigenous knowledge, Mandaya, protected landscape

## Abstract

The Philippine brown deer (*Rusa marianna* Desmarest, 1822) is a tropical cervid increasingly threatened by hunting and habitat loss. It is endemic to the Philippine archipelago, now listed as Endangered under the Philippine Red List and as Vulnerable in the IUCN Red List. This study examined the cultural sensitivities and perspectives of the Mandaya Indigenous peoples through in-depth interviews with 29 Indigenous experts and community observations from Davao Oriental, Philippines. In Mandaya cosmology, the brown deer is known as *Yatag ni Magbabaya*, or a gift from the Creator, and is regarded as an integral part of Mandaya culture and the ancestral lands. The brown deer is a sacred species and an indigenous food source, and its hunting is a Mandaya tradition passed down through generations. Anthropogenic disturbances remain the leading threats against the brown deer, including forest clearing and indiscriminate hunting. Poverty is also a critical driver of hunting pressures, inducing many community members to depend on forest resources and products. The causal layered analysis revealed that human-deer interactions shape local perspectives on conservation, providing insights into the effectiveness of national protection policies. In addition, the futures triangle envisions a sustainable brown deer population to address food security and economic challenges. The research findings call for culturally sensitive conservation strategies and contextualised community education that align with the Mandayas’ traditions and values. Their engagement ensures that conservation goals are met while addressing the community’s socio-economic needs. This study also emphasises the urgent need to reassess the brown deer conservation status to inform protection and management decisions.

## Simple Summary

The present study examines the conservation of the Philippine brown deer (*Rusa marianna* Desmarest, 1822) in Davao Oriental, Mindanao Island, Philippines, with a focus on the perspectives and traditions of the Mandaya Indigenous people. The deer, considered sacred and a cultural symbol, faces declining populations due to poverty-driven hunting and deforestation. Through interviews and observations, the study finds that the Mandaya’s beliefs, traditional practices, and knowledge influence conservation efforts. Collaborative communication, culturally sensitive and future-oriented conservation strategies, and Indigenous-managed protected landscapes are recommended to balance cultural traditions and wildlife protection. Involving Indigenous peoples is crucial for effective and sustainable deer conservation in the Philippines.

## 1. Introduction

The Philippine brown deer (*Rusa marianna* Desmarest, 1822), known in the Cebuano language as *Binaw*, is one of the three endemic deer species in the Philippine archipelago, alongside the Visayan spotted deer (*Rusa alfredi* Sclater, 1870) and the Calamian deer (*Axis calamianensis* Heude, 1888). The genus *Rusa* (Cervidae: Cervinae: Cervini) is distinguished by its rugose, three-point antlers. Both Philippine rusine deer are typical dwarf insular forms, characterised by short legs and antlers [1,2,3].

It is a protected species under the Republic Act No. 9147, otherwise known as the “Wildlife Resources Conservation and Protection Act,” and declared endangered under the Philippine Red List of Threatened Wild Fauna [4,5]. The brown deer is categorised as Vulnerable by the International Union for Conservation of Nature (IUCN) Red List of Threatened Species, assessed over 10 years ago [6]. Prevalent conservation threats include poaching and habitat loss, which have rapidly dwindled the brown deer population across the country [7,8,9].

This cervid was previously widespread across the Philippines, with a distribution range that included Luzon, Samar, Leyte, and Mindanao. As a forest-dependent species, its distribution is now narrowed in the remaining forest ecosystems, and local extinctions have been documented in a few isolated habitats [6,10]. The country’s forestland has decreased significantly from 15 million ha in 2014, when the last IUCN Red List assessment of the brown deer was undertaken, to 7 million ha in 2020 [11,12]. This means that in a span of 6 years, forest cover was reduced at an alarming rate of approximately 46%. The need to reassess the conservation status of the brown deer is now urgent, as it informs protection, management, and conservation decisions at national and international platforms.

Indigenous Cultural Communities (ICCs) and Indigenous Peoples (IPs) are globally celebrated as environmental stewards, playing crucial roles in natural resource management and wildlife conservation [13,14,15]. The same is true for the brown deer, which has been declared as *Pusaka* by the Obu Manuvu in Davao City, Philippines [16,17]. *Pusaka* is an Indigenous tradition of declaring objects sacred, such as the brown deer, which is considered a cultural keystone species [18]. This Indigenous practice translates into conservation actions, protecting species valuable to the community’s history and culture. As such, for endangered species like the brown deer, it is critical to collaborate with ICCs/IPs in effectively implementing community-based conservation approaches.

The term Mandaya is derived from the words “man” or people, and “daya/ilaya” or the upper portion of rivers. They are Indigenous peoples who have settled in the upstream communities of Davao Oriental since the 16th century [19]. The present study explores the cultural sensitivities and perspectives of the Mandaya Indigenous people in Davao Oriental, Mindanao Island, Philippines. Specifically, this paper examines human–wildlife conflict and coexistence in relation to brown deer inhabiting the Mandaya ancestral domain and a Philippine-protected area. Through a futures lens, the study results are intended to propose culturally responsive conservation approaches and strategies to sustain a thriving brown deer population.

## 2. Methodology

### 2.1. Study Sites

**Figure 1:**
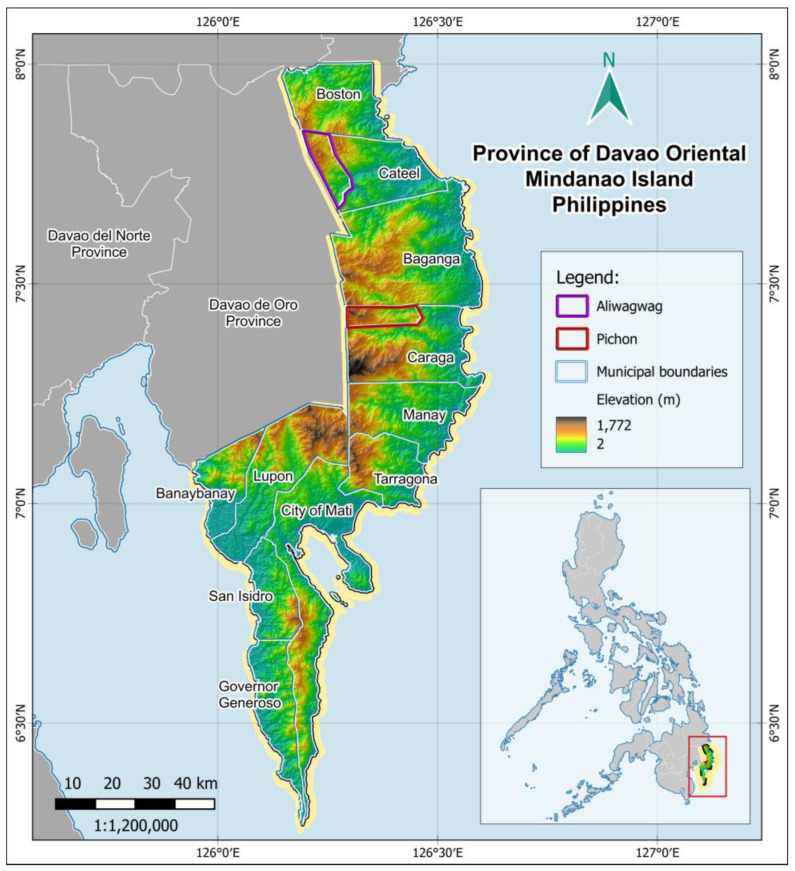
Map of the Study Sites in Davao Oriental, Mindanao Island, Philippines.

Two study sites were selected for this study due to their intact forest ecosystems, which serve as habitats for brown deer and maintain a rich cultural heritage (Figure 1). The two study sites overlap with the ancestral domain of the Mandaya Indigenous Peoples. The first study site is at Barangay Aliwagwag, Cateel, Davao Oriental, Philippines. This village is part of the larger Aliwagwag Protected Landscape (APL), situated within the Eastern Mindanao Biodiversity Corridor. This protected area encompasses more than 10,000 ha of a strict protection zone and 1300 ha of a buffer zone. The protection of APL has been in effect since 2011, as declared through Presidential Proclamation No. 139, the National Integrated Protected Areas System (NIPAS), and the Expanded National Integrated Protected Areas System (E-NIPAS) [12]. Its territory spans the municipalities of Boston and Cateel in Davao Oriental, as well as Compostela in Davao de Oro.

The second study site is in Sitio Caliongan, Barangay Pichon, Caraga, Davao Oriental, Philippines. Forest ecosystems of this sitio, a small rural community or settlement, extend to Mt. Tagubud, also known as Mt. Pandadagsaan or White Peak. This mountain range is part of the Mt. Tagub-Kampalili Complex, an unprotected area in the EMBC, located at the border between Caraga in Davao Oriental and New Bataan in Davao de Oro. This site is a relatively unexplored and under-researched area in terms of biodiversity research and conservation.

### 2.2. Research Design

A qualitative ethnographic research design was employed in this study. Through a qualitative inquiry, the researchers examined the individual experiences of the participants, including the uniqueness, diversity, and meaning of these experiences [20]. Ethnography is a form of qualitative research that collects observations, interviews, and documentary materials to create in-depth and thorough descriptions of various social phenomena [21,22]. This design has been helpful in conservation science, focusing on wildlife and human–wildlife interactions through observations, inquiries, and narrations, especially by Indigenous experts [23]. Specifically, a place-based ethnography was conducted, approaching environmental governance from a geographically bounded perspective [24]. In this study, the social phenomenon of brown deer conservation was investigated from the viewpoint of the Mandaya Indigenous peoples. Fieldwork activities were conducted in situ. First-hand community observations were also undertaken as the first and second authors are residents of Davao Oriental and have been immersed in the Mandaya culture.

### 2.3. Community Interviews

Community interviews were conducted to investigate the cultural sensitivities and perspectives of the Mandaya Indigenous peoples regarding brown deer conservation. Two interview methods were employed: Key Informant Interviews (KIIs) and Focus Group Discussions (FGDs). These methods have been extensively used in community-based wildlife conservation, yielding valuable insights from local communities on how to protect, manage, and conserve natural resources effectively [25,26,27]. All participants were interviewed individually to gather information about their ecological knowledge, current practices in brown deer conservation, the challenges they encountered, and their future perspectives. As a data triangulation technique and to further enhance the quality of data obtained in the KIIs, two FGD sessions were also held. A paper argued that interviewing 10 participants may be sufficient for a qualitative inquiry [27]. It is worth noting that this is just a recommendation rather than a prescription. The number of interviewees largely depends on theoretical saturation, achieved when no new information is obtainable from the interview records [28].

### 2.4. Research Instrument and Participants

A researcher-made interview guide was used to conduct the KIIs and FGDs. This refers to a semi-structured instrument outlining probable questions for the participants. Follow-up questions were also raised to probe the participants’ responses. Some of the questions utilised in this study were based on the Indigenous ecological knowledge inquiry among the Obu Manuvu in Davao City, Philippines [16]. The modified interview guide used in this study underwent expert validation. Three experts in ecology, indigenous studies, and biodiversity conservation were asked to review the instrument before administering it. Revisions were made in accordance with the recommendations of the experts. The instrument was also translated into the Mandaya language to ensure the participants understood the interview. The translation was undertaken by the Mandaya Indigenous Peoples Mandatory Representative of the Municipality of Caraga. The KIIs involved a total of 18 participants, 8 of whom were from Barangay Aliwagwag and the other 10 from Barangay Pichon. These participants include barangay officials, natural resource management officers, park rangers, and indigenous experts, specifically Mandaya leaders and elders. Six participants were engaged in the first FGD session in Barangay Pichon. The second FGD session was conducted in Barangay Aliwagwag with 5 participants. The interview questions and their corresponding translations into the Mandaya language are presented in Appendix A at the end of the manuscript.

### 2.5. Thematic Analysis

The primary analytical method used in this study was thematic analysis. By definition, thematic analysis systematically examines qualitative data and interprets recognisable patterns or themes [29]. This process involves several steps: analysing participant information, organising and preparing the data, thoroughly reading the information, coding the data, developing descriptions and themes based on the codes, and then representing the findings using figures. Finally, the findings are interpreted and discussed to draw meaningful conclusions. The thematic analysis has been employed in uncovering patterns of human–wildlife conflict and local perceptions [30,31,32]. The results of this analysis were then utilised to prepare the Causal Layered Analysis (CLA) and Futures Triangle (FT).

### 2.6. Futures Thinking

Futures thinking is a systematic approach to imagining multiple futures, utilising analytical and foresight tools to understand the past and present, while providing insights into the future [33]. Over recent years, there has been an increasing demand for the application of foresight science in conservation scholarship [34,35]. In this study, futures thinking is employed to critically examine problems in brown deer conservation, drawing on qualitative information from Indigenous peoples and local communities.

The CLA was utilised as an analytical framework in this study. Participants’ responses were analysed in four layers: litany, system, worldview, and narrative and metaphor. Developed in the late 1980s [36], this analysis explains and questions the visible litany or problem based on underlying causes, discourses, and emotive dimensions that are often invisible [37]. This can be illustrated using an iceberg model, with the tip representing the visible problems and challenges in brown deer conservation. The invisible layers deconstruct and provide critical insights into resolving the said challenges.

In conjunction with the CLA, the FT is also used to determine the weight of history, push from the present, and pull from the future to define plausible futures [33,38]. Brown deer conservation, as viewed from this analytical tool, is examined based on historical factors influencing its population dynamics, the present population status and conservation threats, and future perspectives and opportunities. Overall, the futures triangle represents the collective imaginations of participants regarding the future of brown deer conservation, balancing the opposing and interlocking three dimensions.

## 3. Results

### 3.1. Indigenous Knowledge

**Figure 2:**
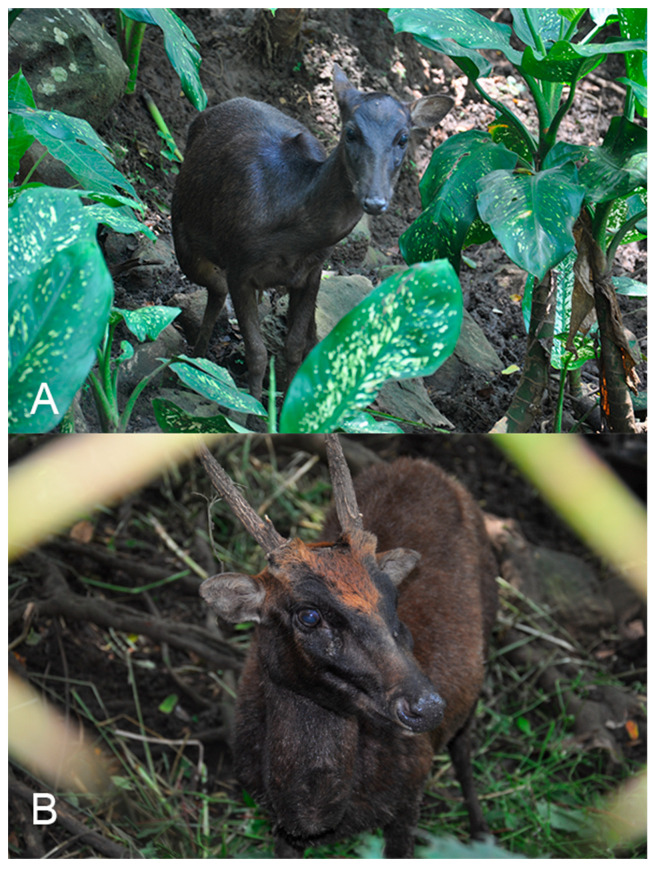
Deer in captivity: (**A**) Female and (**B**) Male at the Philippine Eagle Centre (PEC), Davao City, Mindanao Island, the Philippines.

For the Mandaya people, the brown deer (Figure 2) is known as *Inatyanan* (for the adult female) and *Panga’an* (for the adult male). The juvenile is called *Mal’lakabi*, while *Tal’labuntukan* refers to the male deer with newly grown antlers, and *Tagatul’lian* pertains to the male deer with branching antlers. They recognise the deer to have white spots after birth, which eventually disappear as the deer grows. Adult deer have brown pelage that becomes darker (i.e., dark brown or nearly black) with age. Only the male deer have antlers, which are shed and regrown annually, although the exact timing and natural causes are unknown to the community. The brown deer have a low birth rate and no recorded instances of twins. The community observed no diseases afflicting the brown deer. They believe old age, accidents, predation, and human persecution often cause mortality.

The diet of the brown deer includes a variety of grasses, leaves, shrubs, and seeds. Notably, the deer feeds on rattan fruits (Arecaceae) and is observed to be browsing in certain forest parts, especially during the fruiting season of the rattan plant in December. The community has also identified other plants eaten by the brown deer, such as the *Impatiens platypetala* Lindl., *Homalomena philippinensis* Engl., *Actinodaphne multiflora* Benth., *Ficus* sp., *Macaranga hispida* (Blume) Müll. Arg., *Diospyrus philippinensis* A.DC., *Shorea astylosa* Foxw., *Aquilaria malaccensis* Lam., *Dissochaeta acmura* Stapf & M.L., *Melastoma malabathricum* L., and *Dendrocnide meyeniana* (Walp.) Chew, among others. The majority of these plants are commonly found in the study areas, highlighting the dependence of the forest and wildlife. Historically, when the deer population was still abundant in the region, the species was also noted to encroach on agricultural lands, foraging on crops such as corn (*Zea mays* L.), sweet potatoes (*Ipomoea batatas* (L.) Lam), cassava (*Manihot esculenta* Crantz), and upland rice varieties (*Oryza sativa* L.). There were reports that the deer also feed on ashes, usually observed after *kaingin*, a slash-and-burn or swidden farming system practised by the Mandaya community.

“*Pigakaan nilan sagbot, damilay, mais, kamote, balik balikan nilan kung malapit sang lasang ing pawa mo. Usahay kadamanan da lang kay mukaan man ng mais, kamote usahay posilon da lang*.” IPN03 (They eat different kinds of grasses, corn, and camote in farmlands. If your farm is near the forest, they will always go back to it to eat the crops. Sometimes, it’s the reason why we shoot them.)

In terms of behaviour, the brown deer is a shy and elusive species. They usually move to other areas in response to human disturbance and are described as being wary of humans and dogs. The deer is challenging to catch; thus, managing to hunt one is a sign of masculinity and physical strength for the community. Their movement patterns are also influenced by food availability, as they actively search for food in forest landscapes. As a form of recreation, the deer play with each other and jump around. They were also observed jumping in the air with all their feet off the ground. The deer is a nocturnal species, meaning its physical activities occur primarily at night; however, anecdotal evidence from the community suggests that the deer may also be active during the day. They also make loud noises as a form of communication, especially at night and most notably during a full moon.

“*Kinaiya nilan kalaban nilan yang otaw, ido. Makabaho lang gani mudagan da dayon bisan wapa kaw kitaa*.” INP3 (They don’t like humans and dogs, so as soon as they smell them, they will run fast).

Brown deer habitats are found in the forest interior, far from human habitation. Bedding areas of the brown deer can be found in caves, near cliffs, and underneath fallen tree trunks. They share habitat use with other terrestrial mammals, such as the Philippine warty pig (*Sus philippensis* Nehring, 1886), although interspecific competition has not been documented. Narratives from the community revealed that a juvenile deer was preyed upon by a Philippine eagle (*Pithecophaga jefferyi* Ogilvie-Grant, 1896). Also, there are reports that a Reticulated python (*Python reticulatus* Schneider, 1801) constricts and immobilises the juvenile deer first, then attempts to swallow it. Notably, domestic dogs (*Canis lupus familiaris* Linnaeus, 1758) chase and kill deer, often assisting a human hunter. The deer is poached or hunted by locals for its venison, skin, and antlers.

Overall, the brown deer in Mandaya territories continue to face a population decline. Sustainable resource management should focus on community-based monitoring, habitat restoration, responsible hunting regulations, and environmental education to minimise and manage threats. This is especially important to protect the genetic pool of the deer subspecies found in the study areas, which still requires a more comprehensive taxonomic investigation.

### 3.2. The Deer in the Mandaya Culture

In Mandaya cosmology, the brown deer is referred to as *Yatag ni Magbabaya*, or a gift from the Creator. It is a sacred element of the *yutang kabilin* or the Mandaya ancestral lands. The community believes that a forest spirit guards the brown deer and has declared it a sacred species. The forest is also a sacred ground where they are free to practice their cultural beliefs and traditions. The deer and entire forests are essential to the Mandaya life, culture, and history. Traditional hunters perform the *Panawagtawag*, a community prayer, before embarking on a deer hunting journey. Skilled hunters also claim that the locations of the deer are revealed to them through dreams.

“*Naay ritual sa pagpanghunting. Nakadungog ko ug na-experienced napud nako ky nakakuyog man ko kung manguha ka kinahanglan magdala ka ug buyo, mamaon, linaga na itlog or tuba biya’is, didto ka mag ritual nga mangayo ka, murag mananghid ka sa ilaha nga naa man ka gidala nga halad para tagaan ka. Tested and proven na siya nga makakuha jud*.” IPN02 (There is a ritual in hunting. I heard about and also experienced this because I used to go wildlife hunting. If you want to hunt, you need to bring *buyo*, *mamaon*, hard-boiled egg, and coconut wine, as an offering to the forest spirits to ask for approval to hunt. This has been tested and proven that if you do a ritual, you can catch deer).

**Figure 3:**
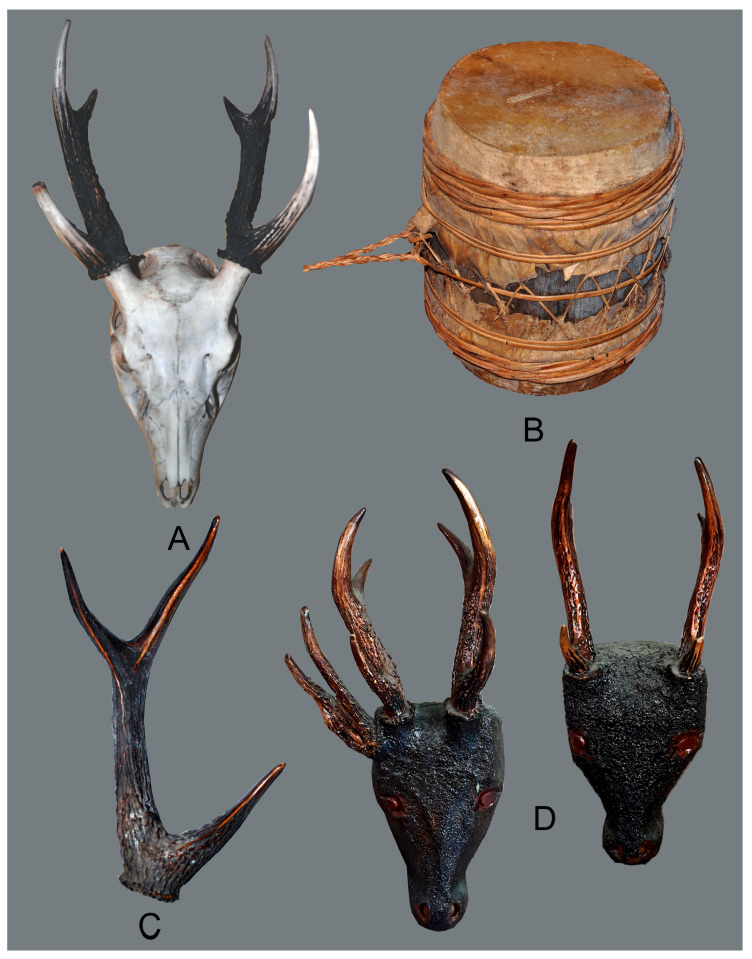
Ethnozoological uses of the Philippine brown deer (*Rusa marianna* Desmarest, 1822) by the Mandaya Indigenous Peoples: (**A**) antler, trophy; (**B**) deerskin or hide, *Gimball*; (**C**) antler, charm; (**D**) antler, house decorations.

Figure 3 illustrates the various ethnozoological uses of deer by the Mandaya Indigenous communities in Davao Oriental. Deer skulls with attached antlers are typically collected as trophies or evidence of their hunting prowess. This relates to their belief that skilled deer hunters are eligible to become *Bagani* or high-ranking warriors. They also keep the deer antlers as home decorations, usually fixed to walls, and use them as racks for hanging clothes, caps, accessories, and other valuable items. When roaming around the forest interior, hunters may find naturally shed antlers. This is a rare find and sighting, so they use these as charms to bring good luck.

In addition, deerskin or hide is also used to craft the *Gimball* or a drum. One side of the drum is prepared with male deer skin, and the other with female deer skin, resulting in distinct sounds. This musical instrument is popularly used in community celebrations. One is the *Kalindugan* Festival, which translates to “foundation,” observed to honour the Mandaya Indigenous peoples’ legal ownership and control over their ancestral lands. The festival is celebrated every October in Sitio Sangab, Barangay Pichon, Caraga, Davao Oriental. Captive brown deer are sometimes displayed during these celebrations. The locals observed that deer faecal droppings influence floral bloom, suggesting that such organic matter may be used as a plant fertiliser.

Deer meat, also known as venison, is a traditional food of the Mandaya people. The meat is often cooked in a freshly harvested, immature bamboo tube, locally known as *Lo’ot.* Deer adobo and soup are also commonly cooked in households. Mandaya dishes with venison are believed to have therapeutic properties. Likewise, the velvet of the deer antlers is scratched, and the powdery residue is mixed with hot water to cure stomachaches. Trees and other plants scratched or eaten by the deer are also believed to have medicinal properties.

“*Pero yang tambal na gusto mo kuwanon yang lidlidin ng sungay nilan tambal yaan, butang ko ngini kahoy ana anaan (bibag idan ng sungay) nilan tambal yaan. Ituyo yaan na bag’idin para kahinlan ng otaw, amo yaan ikatabang sang otaw ng usa. Dili lang makalahong daw unan ing katambalan basta ibag’idan nilan. Tibason mo ngiyan na kahoy kung awon kanmo samad bali gubuhan mo madalihay mahulihan boot pasabot tambal ngiyan ng samad. Kung dili pa kamangon mo ing gamot, ituson mo kung kasaktan kaw ng tiyan kung madayaw kaw sa ato pa tambal sang tiyan, yatry nami yaan*.” IPN05 (For medicinal purposes, you need to find a bark where the deer has frayed its antlers. If a deer frays its antlers on a particular tree or plant, it means that the tree or plant is medicinal. We can’t identify the illness that it can cure until we try it. If you have a wound, pound the outer bark and apply the tree sap to your wound. If you get better, it means the tree is a medicinal remedy for the wound. If you have a stomachache, you can also boil that part of the tree and drink the juice, then you will be healed).

Human-deer interactions have been historically evident in the Mandaya culture. The Mandaya people enjoy seeing the brown deer alive or hearing their calls as part of their recreation. Some households try to keep them as companion animals, although this is rarely successful. Gendered activities were also documented, with men in charge of hunting and processing the meat, while women prepared the meals for the family. Hunting skills are also passed down to different generations, specifically from fathers to their sons, by engaging them in hunting activities early on.

### 3.3. Population Status and Threats

**Figure 4:**
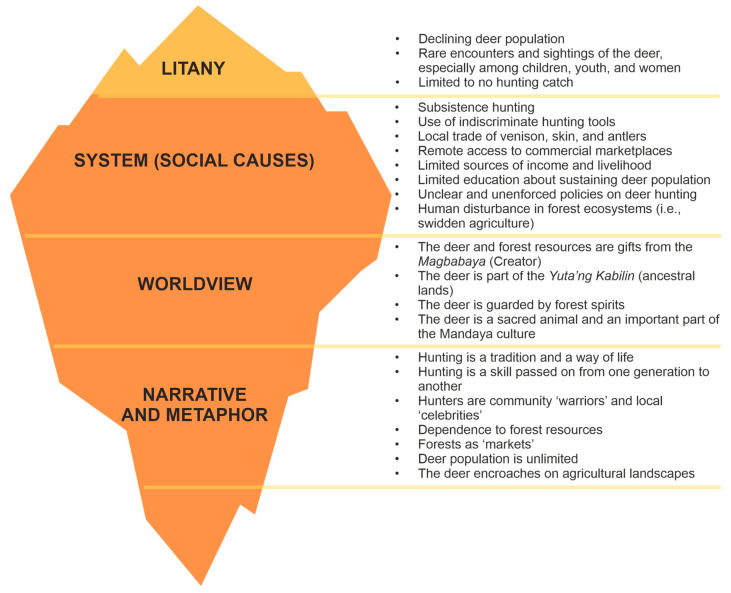
Causal Layered Analysis (CLA) on the Conservation of the Philippine Brown Deer (*Rusa marianna* Desmarest, 1822) in the Mandaya Ancestral Lands in Davao Oriental, Philippines.

Figure 4 presents the CLA, revealing that the brown deer population is already declining in their natural habitats, as evidenced by the limited hunting catch. Hunters have compared that around ten years ago, it was relatively easy to hunt deer. One hunter even claims to have captured eight deer in one trip, which is nearly impossible nowadays. The women, youth, and children claim they have not encountered or seen the brown deer, despite living near the forests.

Hunting remains an indispensable practice despite prohibitions based on Philippine wildlife protection laws. In this context, hunting is primarily for subsistence rather than profit or income generation. Different hunting tools, such as snare traps and *bal’latik* (sharpened bamboo sticks), are used in this practice. Aside from the brown deer, the hunters also target the warty pig, python, Philippine flying lemur (*Cynocephalus volans* Linnaeus, 1758), Yellow-headed water monitor (*Varanus cumingi* Martin, 1839), and other non-volant mammals and birds. The traps are installed in the forest interior but are sometimes forgotten by the hunter, causing unaccounted and undocumented wildlife mortality. They also practice *pagpangayam*, a hunting technique with the aid of domestic dogs. The dogs are groomed and trained by their owners to hunt and kill deer and other wildlife. In more recent times, hunters use guns, which they claim to be more appropriate, as these tools kill the deer immediately, allowing them to collect the body in real time. All these techniques are indiscriminate as they capture juvenile, female, and male deer individuals. A hunter reports having experienced pursuing a pregnant deer.

“*Awon da kalahian doon kay tungud madaig da tigkamang, ikaduha galayo da kay gapakabaho da ng gasolina ng sakyanan, awon yusod ng logging, chainsaw madungog da, syempre mangikyas gayud yaan silan awon pero malayuay da tagsara doon ing malapit*.” IPN03 (There are changes in the brown deer population due to the increasing number of hunters compared to before. Secondly, the deer leave the area whenever they encounter loggers, hear vehicles and chainsaws, or smell gasoline. There are rare sightings now).

Poverty and wildlife hunting are interlinked. The limited livelihood options and narrowed access to socio-economic services are critical drivers of the local trade of venison, antlers, and deerskin. The venison is sold locally for PhP350.00 to PhP400.00 per kilogram. With the unpaved farm-to-market roads, the community is compelled to depend on the forest as their local ‘market,’ where they can hunt and obtain food. A few local eateries, known as *karinderya*, were also reported to be selling deer *adobo*, a popular Filipino dish typically cooked with soy sauce, vinegar, and garlic, at approximately PhP100.00 per serving. This dish is no longer available at these eateries during recent visits, apparently due to persistent reminders from the Department of Environment and Natural Resources (DENR). Deer skins and antlers are sold to local collectors, revealing a more complex wildlife trade value chain that remains largely undocumented. Other anthropogenic disturbances, such as swidden agriculture, further drive deer populations into the fragmented and reduced forest cover.

“*Ngining usa tungud man gud ng kalayo nami sang baba sang palengke maipit da kami way mamasud-an manghunting gayud kami saan para paga-otan, kay way pamasahe pagkadto sang palengke agaw mapugos da lang kami sian*.” IPN03 (Because we are far from the market, the cost of fare to go there is too expensive; therefore, if we have no viand, we are forced to hunt for our family to eat).

Community education initiatives are intensive in Barangay Aliwagwag, which is a stakeholder of the APL. However, Barangay Pichon is currently not protected, making wildlife protection campaigns more challenging. Brown deer conservation is not explicitly mentioned in most community education and public awareness initiatives, which are often focused on the Philippine eagle. There is limited information dissemination about sustaining the brown deer populations in the Mandaya ancestral domain. Also, most protection, management, and conservation policies are unwritten. Thus, their enforcement is often insufficient and inconsistent.

In the Mandaya worldview, the brown deer is a sacred gift from the Creator, known as *Yatag ni Magbabaya.* They believe the species is a God-given blessing that should be used and consumed by humans. The brown deer is deeply intertwined with the Mandaya culture and is considered an integral part of the *yuta’ng kabilin* or ancestral lands. This human–wildlife connection signifies that the deer is a vital aspect of the Mandaya cultural identity, and its presence and abundance in the sacred grounds must be preserved. The community also believes that the brown deer is owned and guarded by forest spirits, which means that any form of disrespect to the species is unacceptable. Some hunters even assert that successful hunting relies on receiving permission from the forest spirits.

“*Kung awon tag-iya kanilan mamadaman man gayud kung hantingon, siguro binuhat silan ng Ginoo o Magbabaya na para isab kanato.*” (If the deer has an owner, I think he will get angry if someone hunts it. I believe the deer was given to us by God, for us to use it).

The narratives and metaphors are drawn out from the emotional and interpretive depths of the personal experiences of the Mandaya people. They have revealed that hunting is a tradition that has been practised for many generations. It is a way of life that cannot be easily stopped, even with national hunting prohibition policies in place. The community highly regards accomplished hunters and fondly refers to them as local celebrities. While the brown deer is challenging to catch due to its elusive behaviour, hunting requires special skills that characterise a warrior. Many hunters are also community defenders, known as *Bagani*. The hunting tradition is preserved across generations. Hunter-fathers involve their male children in hunting activities at a young age. Hunting is a socially acceptable practice, with the brown deer population described as unlimited. This means that, despite the significant hunting pressure, they believe that the deer population will never be decimated. They also regard the forest as a traditional marketplace, a source of Indigenous food items necessary for the community’s survival. For them, deer hunting is essential for regulating the wildlife population and preventing encroachment on agricultural lands.

### 3.4. Conservation

In the APL, natural resource management and biodiversity conservation are undertaken by the Protected Area Management Office (PAMO), which is governed by the Community Environment and Natural Resources Office of Baganga. The PAMO supervises several park rangers at the forefront of the natural landscape protection mission. In 2024, there are 11 park rangers, comprising 8 males and 3 females. They participate in demarcating and delineating the boundaries of the protected area and assist in conducting the Biodiversity Assessment and Monitoring System. Research and conservation programs, projects, and activities are regularly carried out with the same personnel involved. A multi-sectoral policy and decision-making body oversees these, namely the PAMB, which was duly created pursuant to the mandates of Republic Act No. 7586, also known as the NIPAS Act. PAMB is composed of the local government units, Indigenous peoples’ representatives, non-government organisations, and academic institutions, among others. This composition allows multiple perspectives to collectively discuss and decide on APL policy matters.

On the other hand, the forest landscapes proximal to Barangay Pichon remain unprotected on a national scale. They are not included in the NIPAS or the new declarations under the Republic Act 11038, otherwise known as the E-NIPAS. This area is governed by the Mandaya people per the Certificate of Ancestral Domain Title 01 (CADT-01) issued by the National Commission on Indigenous Peoples, in accordance with Republic Act No. 8371, also known as the Indigenous Peoples Rights Act. The community is mandated to promulgate an Ancestral Domain Sustainable Development and Protection Plan, which will cover the description of the domain, the community’s situation, development priorities, and investment plans. Wildlife protection and environmental management initiatives are crucial to the same strategy. However, at present, most of the deer protection and hunting regulation policies remain unwritten. These policies are typically promulgated by the Mandaya Council of Elders and Leaders, commonly referred to as the *Limpong ng Mangkatadong*. The *bal’law bal’law*, or conversation, is also practised for discussing community concerns or to announce conservation policies.

At present, there is no captive breeding program for brown deer within the locality. Some people keep a few individuals within their backyards, but the purpose for this remains unclear—either for farming or recreation. These artisanal facilities are often not managed well, with the brown deer being fed with locally available forage. No species introduction efforts for the brown deer have been made in these areas.

The participants shared several policy recommendations to conserve the brown deer in their ancestral lands. Foremost, they emphasised the need to educate the public about the deer and its importance to the environment. Lectures about wildlife protection must also be undertaken by the academe, the DENR, and other government agencies. This is a critical step in enforcing deer protection policies, especially now that they perceive the deer population is already declining. While they argue that hunting for customary use is allowed under the IPRA Law, they are willing to implement a “no hunting” policy. Others also suggested regulating current hunting practices. They want to prohibit the use of indiscriminate tools and manage feral dogs to avoid irresponsible brown deer mortalities.

*“Yagka teamwork yang trese (13) ka sitios na dida ilabtan, suma ng balaod ng gobyerno. Yagpasibaw da ng balaod ing gobyerno na tagaan yang chieftain ng isa ka balaod na dili paghilabtan.”* IPN05 (The thirteen villages agreed not to hunt/disturb deer according to our government laws; the government gives the directive to the chieftain not to hunt the brown deer).

The steady influx of tourists to the Aliwagwag Falls Eco Park, featuring the tallest cascading waterfalls in the Philippines, has provided economic incentives to the locals. The community aims to explore additional economic and livelihood opportunities based on this ecotourism model. They also remarked that the deer, a charismatic species, could be used for ecotourism. In addition, entitled “environment heroes,” the roles of the park rangers are indispensable in the conservation mission. There is a local clamour to formalise forest guarding as a profession, increase their numbers, intensify their capacity, and provide more incentives to make it an attractive career.

### 3.5. Futures of Deer Conservation

**Figure 5:**
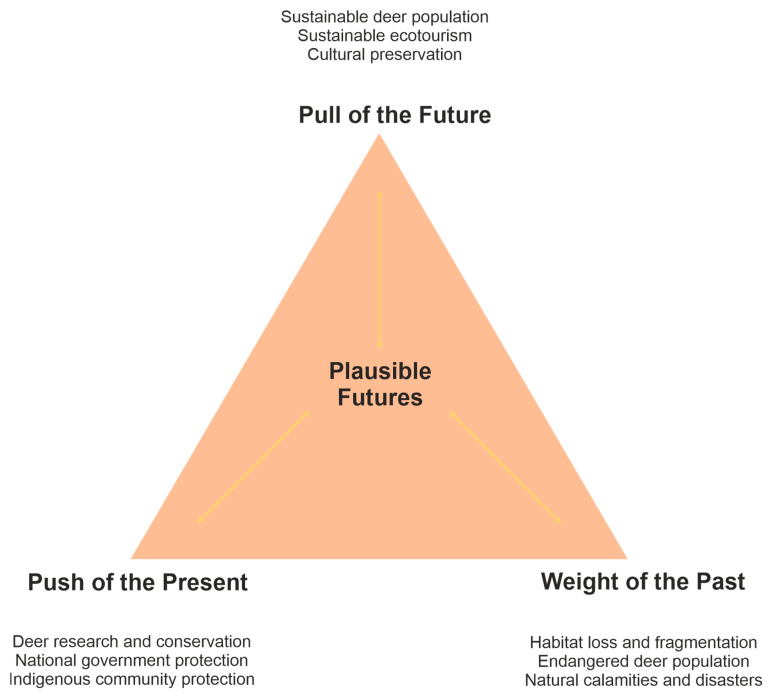
Futures Triangle for the Conservation of the Philippine Brown Deer (*Rusa marianna* Desmarest, 1822).

Figure 5 illustrates the FT on brown deer conservation, balancing three dimensions: the pull of the future, the push of the present, and the weight of the past. The participants envision a sustainable future for the deer, aiming to increase the dwindling deer population by actively participating in the conservation mission. They are also committed to adhering to hunting bans and regulatory policies that allow the species to recover. Further, they emphasise the importance of sustainable consumption, particularly if the deer population becomes stable. In this way, the community can preserve its cultural practices and address food security challenges that were magnified during the COVID-19 pandemic.

One plausible initiative is the establishment of Indigenous community conservation areas within CADT-01 to protect the brown deer and its natural habitat. Current circumstances significantly influence the abovementioned future scenarios. The perceived decline in the brown deer population motivates the community to pursue protection measures and strengthen existing conservation initiatives. Landscape protection laws promulgated by the national government are critical to deterring wildlife crimes. Similarly, Indigenous protection policies effectively address the challenges of in situ conservation.

These efforts aim to address the past issues that have impacted the brown deer population. One of these is a tropical cyclone, *Bagyong Pablo* (International Name: Bopha), that struck the province of Davao Oriental in 2012. Accordingly, the super typhoon impacted the forest vegetation and the wildlife that depend on it, although no scientific records have been published regarding this impact. Additionally, the intensive logging activities prevalent in many parts of the country, including the study areas, in the 1970s, led to the loss and fragmentation of deer habitats. The indiscriminate hunting of brown deer further exacerbated this.

## 4. Discussion

A multitude of literature underscores the critical roles of Indigenous peoples in wildlife conservation worldwide. Environmental stewardship by Indigenous peoples and local communities has been instrumental in long-term biodiversity conservation [39,40,41]. Indigenous knowledge systems are now increasingly recognised in global science-policy platforms [42,43,44]. In Canada, an emerging urban conservation governance framework is being developed, grounded in Indigenous knowledge systems, rights, and responsibilities, with a focus on biocultural priorities [45]. The current state of brown deer conservation in the Philippines is summarised through the analysis of strengths, weaknesses, opportunities, and threats (SWOT) (Table 1).

Community-based conservation is central to the efforts to protect and repopulate the Critically Endangered Philippine Eagle, spearheaded by the Philippine Eagle Foundation, a non-government organisation. They have been working with the Obu Manuvu people and other Indigenous groups in Mindanao, harnessing their philosophies and practices to establish long-term strategies for eagle protection and forest management [18,46,47]. With the help of the Obu Manuvu people, a study recorded a warty pig wallow, which served as a drinking well for the brown deer and other species [8]. This highlights the critical roles of Indigenous peoples in wildlife monitoring initiatives.

The same strategy could be explored for the deer, although it is still in its early stages and requires extensive study [10,16]. Human–wildlife coexistence must be emphasised rather than focusing on conflict [48]. The current study argues that human-deer interactions in the Mandaya ancestral domains could be coadaptive and mutually beneficial, although urgent efforts must be undertaken to prevent conflicts.

The Indigenous Peoples Rights Act in the Philippines stipulates the sustainable traditional resource rights of the Indigenous peoples [49]. Section 9A of the same law enshrines the responsibility of the IPs to maintain ecological balance by protecting wildlife and natural resources in their ancestral domains. They are mandated to use, manage, protect, and conserve their land and water resources sustainably, including their hunting grounds. This provision recognises hunting as a sacred tradition shared by many Indigenous communities throughout the country. Specifically, deer hunting, consuming venison, and using deer derivatives (i.e., antlers and skin) are permitted for customary practices. Given the diminishing cultural traditions among diverse ethnolinguistic groups, it is essential to establish mechanisms to preserve them [50,51,52]. In this context, cultural preservation must be approached in a manner that also facilitates wildlife conservation. There is a need to promote conservation policies, such as hunting bans, while also allowing cultural practices to be sustained.

The present study reports that the cultural sensitivities and perspectives of the Mandaya people in Davao Oriental play a crucial part in conserving the brown deer. Their worldview, belief systems, traditions, and practices have a direct impact on the stability of deer populations. Moreover, the Mandaya Indigenous ecological knowledge, learned through their historical familiarity with the forest landscapes, is pivotal to undertaking conservation efforts in their ancestral domain. The Mandaya cosmology declares the deer to be an indispensable part of the Mandaya ancestral lands and a gift from the Supreme Being, *Magbabaya.* Being a gift from the Creator, the community believes that the brown deer must be utilised for customary practices, primarily as a food source. Otherwise, it becomes disrespectful to the giver. Anecdotes from the participants also highlighted that the brown deer population is unlimited and could not be overhunted. This relates to cornucopianism, the belief that natural resources are abundant [53]. Community education and public awareness must address this way of thinking and let them realise the limits of environmental exploitation. *Bayok*, a traditional communication tool of the Mandaya, may be utilised to promote environmental awareness and responsibility [54].

The brown deer is a cultural keystone species and a charismatic species for the Mandaya people. They perceive it as a sacred species and, thus, must be respected. A deep cultural regard for the species translates to conservation value. This relates to the *Pusaka* philosophy of the Obu Manuvu people in Davao City, Philippines, which has been an effective means of recruiting local conservation champions to lead and participate in the ongoing mission to protect the brown deer and the forest. *Pusaka*, a traditional practice of declaring sacred objects, translates to conservation initiatives for the brown deer, in fear of losing a part of their culture [16,17,18]. The *lapat* system of the Isneg and Tingguian people in the Northern Philippines imposes a cultural taboo and prohibits the exploitation of natural resources in designated areas [55]. The participation of Indigenous peoples has also proven pivotal in addressing raptor population reduction and deforestation in the Sierra Madre [56]. By recognising and supporting these approaches, conservation efforts can become more inclusive and culturally grounded, ensuring the protection of biodiversity and the preservation of Indigenous heritage.

The absence of bioecological research and conservation studies limits the development of policies that are sensitive and responsive to the cultural context of the study areas. This underscores the criticality of cultural perspectives and sensitivities in the success of protection, management, and conservation initiatives. Community attitudes, beliefs, and values inform deer management in Scotland [57]. Social-ecological relationships were also found to maintain and support biodiversity through cross-disciplinary research and conservation involving local communities in China [58]. A study highlighted the need to incorporate the Andean worldview and ancestral knowledge into water management, particularly by integrating the core symbols of *Chakana.* This cosmological symbol illustrates equilibrium and interconnectivity [59]. In the Philippines, attitudes, norms, and perceived usefulness toward urban trees positively influence community intentions to partake in conservation initiatives [60]. Likewise, local perceptions, values, and conservation attitudes are also crucial determinants in conserving the Red-backed sea eagle (*Haliastur indus* Boddaert, 1783) [61]. In addition to this qualitative report, quantitative surveys will be conducted to investigate community perceptions and help develop a culture-based conservation model for the deer. The overarching aim is to build up research data and prioritise deer conservation.

Although the brown deer is one of the largest mammals on the Mindanao forest floor, alongside the warty pig, its research and conservation have progressed slowly. There is a wide gap in the biological and ecological knowledge of the deer, which delimits science-based conservation approaches. The latest known Philippine deer monograph was published in 1993, which contained information only on captive populations, including food preferences, female reproduction, and rearing techniques [62,63,64]. A few books have been published successively, necessitating intensive research to enhance current knowledge about deer [6,65,66,67,68].

The brown deer may be declared an umbrella or flagship species for conservation. A similar model was applied in conserving the Cheetah (*Acinonyx jubatus* Schreber, 1775), which directly or indirectly benefited area protection networks [69]. The intention is to address the ever-increasing anthropogenic threats that negatively impact the deer population and shrink the forest ecosystems. In this regard, conserving the brown deer also means protecting the forest and the wildlife that depends on it. On a national scale, efforts to safeguard the deer remain suboptimal. One of the nearest wildlife centres to the study sites is the Philippine Eagle Centre (PEC), which is primarily dedicated to protecting the Philippine national bird. The centre houses several brown deer individuals for educational, research, and conservation purposes [10,46]. There are other facilities across the country, but most are primarily intended for tourism rather than conservation. A Wildlife Rescue Centre was also established in 2011 by the DENR Regional Office XI in collaboration with the Local Government Unit of Tagum City [70]. This serves as a temporary shelter and provides medical interventions to rescued wildlife species. These ex situ wildlife conservation facilities are operational but insufficient, as they cover only a small portion of the vast Davao region landscape.

Currently, the brown deer is not listed as a priority species in the Association of Zoos and Aquariums, the European Association of Zoos and Aquaria, or the World Association of Zoos and Aquariums. This underscores the absence of international conservation efforts for the species. As a result, local initiatives in the Philippines are presently the country’s main hope, providing crucial information that could inform future international conservation efforts.

One promising approach to democratising brown deer research and conservation efforts amidst insufficient funding and ambiguity in prioritisation is to involve Indigenous Peoples and Local Communities. This is best achieved by providing tangible economic benefits to those involved in the conservation work and recognising forest guards and park rangers as a formal career. Deer hunting tourism has been proven to bring socio-economic benefits and yield revenues, often used to support forest and wildlife conservation [71,72]. Although this is currently not viable in the study areas due to the decline in deer populations, ecotourism remains an option. As the community suggests, ecotourism activities involving the deer may be explored to optimise community involvement.

## 5. Conclusions

The conservation of the Philippine brown deer (*Rusa marianna* Desmarest 1822) is a multifaceted challenge that requires a collaborative approach, integrating the knowledge and practices of Indigenous peoples with science-based conservation strategies. Implementing Indigenous community conservation areas represents a promising direction for safeguarding the brown deer population and its forest habitats. However, contemporary conservation interventions are no longer sufficient to ensure the sustainability of the deer population. There is a need to rethink conservation, particularly for species that are subject to commercial exploitation while also holding cultural significance. Addressing this challenge necessitates both economic reforms in natural resource management and the implementation of advanced bioecological solutions, such as habitat regeneration and ex situ breeding programs. Enhancing protected area management plans that involve Indigenous peoples and local communities is also necessary, taking into account their cultural sensitivities and perspectives. This study positions the Mandaya people in Davao Oriental as environmental stewards based on their historical and cultural interactions with deer and other wildlife. As the community actively engages in protection efforts and adheres to regulations, there is hope for reversing the population decline exacerbated by past environmental challenges and unsustainable practices. Moreover, acknowledging the critical role of Indigenous peoples in biodiversity conservation can pave the way for more effective and culturally sensitive management practices. By fostering partnerships among Indigenous peoples and local communities, government bodies, and conservation organisations, and leveraging Indigenous knowledge and rights, it is possible to create a sustainable framework for protecting the deer and its ecosystem. Overall, the futures will be defined by a commitment to ecological stewardship, cultural preservation, and promoting sustainable practices that benefit wildlife and human communities. Careful monitoring and adaptive management will be necessary to ensure that these efforts yield positive outcomes for the species and the landscapes they inhabit.

## Figures and Tables

**Table 1:** SWOT Analysis of the Conservation of the Philippine Brown Deer (*Rusa marianna* Desmarest, 1822) in the Mandaya Ancestral Domains in Davao Oriental, the Philippines.

Strengths	Weaknesses
Existence of strong indigenous knowledge systems and cultural traditions that support wildlife conservation and sustainable resource use. Deep cultural connections and spiritual significance are attributed to the brown deer, fostering intrinsic motivation for conservation. Proven effectiveness of collaborative conservation models involving IP communities and government institutions with other threatened species in different regions in the Philippines. Presence of legal frameworks (i.e., RA 9147 and RA 8371) that recognise natural resource management and protection of indigenous rights.	Sporadic scientific research on the biology and ecology of the brown deer in their natural environment. Limited local facilities, equipment, human resources, and professional expertise dedicated to brown deer conservation. Absence of formal recognition of local conservation champions and the inadequate economic incentives for conservation services. Insufficient funding and ambiguity in wildlife research and conservation prioritisation. Loss of traditional and indigenous ecological knowledge due to cultural shifts and modernisation.
Opportunities	Threats
Integration of indigenous and scientific knowledge in conservation policymaking and management. Development of community-led ecotourism initiatives to provide economic incentives for conservation. Declaration of the brown deer as a flagship and symbolic species for wildlife conservation and natural resource management. Implementation of educational programs leveraging traditional tools (e.g., *bayok* and *bal’law bal’law*) to transform perceptions about resource limits and conservation necessity. Enhancement of social, biological, and ecological research to build robust community-centred conservation models.	Ongoing brown deer population decline and habitat loss due to anthropogenic pressures (e.g., hunting and timber poaching). Emergence of conflicts between conservation laws (e.g., hunting bans) and indigenous cultural practices, if not properly integrated. Influence of broad social, economic, and political factors potentially affecting community involvement and conservation priorities. Continued loss of indigenous ecological knowledge and cultural practices that underpin sustainable natural resource management. Sporadic in situ and ex situ conservation strategies for threatened Philippine wildlife due to budget constraints and shifting priorities.

## Data Availability

The datasets presented in this article are not readily available due to privacy and confidentiality agreements that protect the identity of the indigenous participants. Requests to access the datasets should be directed to villegas@ftz.czu.cz (J.P.V.).

## Abbreviations

The following abbreviations are used in this manuscript:

APL	Aliwagwag Protected Landscape
BMB	Biodiversity Management Bureau
CADT	Certificate of Ancestral Domain Title
CLA	Causal Layered Analysis
DAO	DENR Administrative Order
DENR	Department of Environment and Natural Resources
E-NIPAS	Expanded National Integrated Protected Areas System
EMBC	Eastern Mindanao Biodiversity Corridor
FGDs	Focus Group Discussions
FMB	Forest Management Bureau
FT	Futures Triangle
ICCs	Indigenous Cultural Communities
IPRA	Indigenous Peoples Rights Act
IPs	Indigenous Peoples
KII	Key Informant Interviews
LGU	Local Government Unit
NIPAS	National Integrated Protected Areas System
PAMB	Protected Area Management Board
PAMO	Protected Area Management Office
SWOT	Strengths, Weaknesses, Opportunities, and Threats

## Appendix A. Interview Guide

1. What do you know about the Philippine brown deer? (*Nyanang kyakasayodan mo sam sol’law na osa disim Banwa ng Pilipinas*)
  - Morphology (*Pagtoon nang kal’lawas aw kaymo nam butang aw bowi*)
  - Diet and feeding (*Pyagakaan o pagkaan*)
  - Territory and habitat (*Paguya*)
  - Reproduction (*Pagdagdag, pagpanganak, pagkadaig*)
  - Behaviour (*Kinaiya*)
2. What are the cultural beliefs about the Philippine brown deer in your indigenous community? (*Nyanang kyakasayudan ta bain nam kanatun kinaiyahan na pagtoo bain sam sol’law na osa nam Pilipinas?*)
3. What are the ethnozoological uses of the Philippine brown deer in your indigenous community? (*Nyanang ‘ethnozoological’ kad’yawan na kyakaatag sang kanatun komunidad nang sol’law na osa ning kanatun banwa nang Pilipinas?*)
4. What is the population trend of the Philippine brown deer in the CADT-01 over the last 30 years? 20 years? 10 years? (*On-nono ing kadaig ng sol’law na osa nam Pilipinas sang CADT-01 disti tol’lompol’lo katuwig?dowampol’lo katuwig? sampol’long ka tuig?*)
  - Increasing (*Gadugang*)
  - Decreasing (*Yagaka katabayda o pyagkakamangan*)
  - Stable (*Madakmol’l o madaigay o ol-la*)
5. Why do you think there are changes in the Philippine brown deer population? (*San tanaw mo nanga al’lon kaisaban nam popolasyon nang sol’law na osa nam Pilipinas?*)
6. What conservation threats concerning the Philippine brown deer have you observed or documented? (*Nyanang iinang mayo na pamaagi antak di mawal’la ing sol’law na osa na kamayo ikita aw pagsol’lat o dolomento?*)
7. What protection, management, and conservation measures and policies are implemented in your indigenous community? What do you think is being done or should be done? (*Nyanang iinang mayo disining kamayo komunidad, na mga pamaagi, san tanaw mo na iinang o inangun antak di matiwas ing mga sol’law na osa?*)
8. What are the gender roles of women, men, and youth in conserving the Philippine brown deer? (*Nanang katungdanan nam kabobayan, kausgan, aw mga sogno antak di mawal’la ing sol’law na osa?*)
9. What are your policy suggestions and recommendations to protect, manage, and conserve the Philippine brown deer? (*Nanang polisiya na aka ambag mo aw sugyot mo antak kabantayan na di matiwas yang sol’law na osa?*)
10. What are your future perspectives on the conservation of the Philippine brown deer? (*Nanang dyudumdum mo o panlantaw mo na mad’yaw inangun antak di matiwas in sol’law na osa?*)

## References

[1] Leslie D.M. (2011). *Rusa unicolor* (Artiodactyla: Cervidae). Mamm. Species.

[2] Geist V., Geist V. (1998). Three-pronged Old World deer. Deer of the World: Their Evolution, Behaviour, and Ecology.

[3] Heckeberg N.S., Melletti M., Focardi S. (2025). The Systematics of Cervidae. Deer of the World: Ecology, Conservation and Management.

[4] (2019). [DAO 09] Department of Environment and Natural Resources Administrative Order 2019–09. Updated National List of Threatened Philippine Fauna and their Categories. https://elibrary.bmb.gov.ph/elibrary/wp-content/uploads/2023/05/dao2019-09.pdf.

[5] [DENR–BMB] Department of Environment and Natural Resources—Biodiversity Management Bureau (2020). Philippine Red List of Threatened Wild Fauna.

[6] MacKinnon J.R., Ong P., Gonzales J. *Rusa marianna*. The IUCN Red List of Threatened Species 2015, e.T4274A22168586. https://www.iucnredlist.org/species/4274/22168586.

[7] Tanalgo K.C. (2017). Wildlife hunting by Indigenous People in a Philippine protected area: A perspective from Mt. Apo National Park, Mindanao Island. J. Threat. Taxa.

[8] Villegas J.P., Rosales J.R., Tampos G.G., Ibañez J.C. (2023). Inventory and abundance of non-volant mammals and birds in the unprotected regions of the Mount Apo range, Philippines. J. Threat. Taxa.

[9] McShea W.J., Ilyas O., Li S., Duarte J.M.B., Melletti M., Focardi S. (2025). Conservation of Deer in South America and Asia. Deer of the World: Ecology, Conservation and Management.

[10] Villegas J.P., Rosales J.R., Verzosa R.C., Ibañez J.C., Ceacero F., Melletti M., Focardi S. (2025). Philippine Brown Deer (*Rusa marianna* Desmarest, 1822). Deer of the World: Ecology, Conservation and Management.

[11] [DENR–FMB] Department of Environment and Natural Resources—Forest Management Bureau (2014). Philippine Forestry Statistics 2014.

[12] [DENR-FMB] Department of Environment and Natural Resources—Forest Management Bureau (2023). Philippine Forestry Statistics 2023.

[13] Nikolakis W., Gay V., Nygaard A. (2023). The ‘environmental stewardship-health nexus’ among Indigenous Peoples: A global systematic literature review. Wellbeing Space Soc..

[14] Palita S.K., Panda D., Nayak J.K. (2023). Indigenous communities and biodiversity conservation: An Indian perspective. Sci. Cult..

[15] Sze J.S., Childs D.Z., Carrasco L.R., Fernández-Llamazares A., Garnett S.T., Edwards D.P. (2023). Indigenous Peoples’ lands are critical for safeguarding vertebrate diversity across the tropics. Glob. Chang. Biol..

[16] Villegas J.P., Ibañez J.C., Cabrido C.K.T. (2022). Abundance and distribution of the Philippine Brown deer (*Rusa marianna* Desmarest, 1822) in the Obu Manuvu ancestral domain, Mindanao Island, Philippines. Acta Biol. Univ. Daugavp..

[17] Bauyot M.F., Villegas J.P., Asaias V.E. (2024). Gender roles of Obu Manuvu women and leaders in the conservation of the Philippine brown deer *Rusa marianna* Desmarest, 1822. Palawan Sci..

[18] [TUOMTC] The Unified Obu Manuvu Tribal Council (2017). Caring for Pusaka.

[19] Nabayra E.S. (2014). Cosmology of the Mandaya. AghamTao.

[20] Sevilla-Liu A. (2023). The theoretical basis of a functional-descriptive approach to qualitative research in CBS: With a focus on narrative analysis and practice. J. Context. Behav. Sci..

[21] Reeves S., Peller J., Goldman J., Kitto S. (2013). Ethnography in qualitative educational research: AMEE guide No. 80. Med. Teach..

[22] Creswell J.W. (2014). Research Design: Qualitative, Quantitative, and Mixed Methods Approaches.

[23] Kiik L. (2018). Wild-ing the ethnography of conservation: Writing nature’s value and agency in. Anthropol. Forum.

[24] Thaler G.M. (2021). Ethnography of environmental governance: Towards an organizational approach. Geoforum.

[25] Oduor A.M. (2020). Livelihood impacts and governance processes of community-based wildlife conservation in Maasai Mara ecosystem, Kenya. J. Environ. Manag..

[26] Mosse M.N., Odadi W.O., Kibue G.W. (2024). Anthropogenic threats to crocodiles, and the level and sociodemographic determinants of their utilization in lower river Tana basin, Kenya. Trop. Conserv. Sci..

[27] Muellmann S., Brand T., Jürgens D., Gansefort D., Zeeb H. (2021). How many key informants are enough? Analysing the validity of the community readiness assessment. BMC Res. Notes.

[28] Rahimi S., Khatooni M. (2024). Saturation in qualitative research: An evolutionary concept analysis. Int. J. Nurs. Stud. Adv..

[29] Squires V. (2023). Thematic Analysis. Varieties of Qualitative Research Methods.

[30] Alam R., Nayak D. (2024). Examining human-wildlife conflict and management strategies in Indian protected areas: Evidence from Jim Corbett tiger reserve. Hum. Dimens. Wildl..

[31] Blackie I.R., Gaodirelwe I., Masole C. (2024). Wildlife killer instincts: Human wildlife conflict and fatal incidents in Botswana. Cogent Soc. Sci..

[32] Mupunga P., Shoko J. (2024). Local community perceptions on human–wildlife interactions in the face of climate variability: A case of Nyaminyami community, Zimbabwe. Front. Sustain. Tour..

[33] Asian Development Bank (2020). Futures Thinking in Asia and the Pacific: Why Foresight Matters for Policy Makers. https://www.adb.org/sites/default/files/publication/579491/futures-thinking-asia-pacific-policy-makers.pdf.

[34] Ednie G., Kapoor T., Koppel O., Piczak M.L., Reid J.L., Murdoch A.D., Cook C.N., Sutherland W.J., Cooke S.J. (2022). Foresight science in conservation: Tools, barriers, and mainstreaming opportunities. Ambio.

[35] Mahajan S.L., Tanner L., Ahmadia G., Becker H., DeMello N., Fidler R., Harborne A.R., Jagadish A., Mills M., Cairney P. (2023). Accelerating evidence-informed decision-making in conservation implementing agencies through effective monitoring, evaluation, and learning. Biol. Conserv..

[36] Inayatullah S. (1998). Causal Layered Analysis: Poststructuralism as method. Futures.

[37] Inayatullah S. (2022). Causal layered analysis: Theory, conceptual framework and method. CLA 3.0—Thirty Years of Transformative Research.

[38] Inayatullah S. (2023). The futures triangle: Origins and iterations. World Futures Rev..

[39] Pelletier J., Gélinas N., Potvin C. (2019). Indigenous perspective to inform rights-based conservation in a protected area of Panama. Land Use Policy.

[40] Dawson N.M., Coolsaet B., Sterling E.J., Loveridge R., Gross-Camp N.D., Wongbusarakum S., Sangha K.K., Scherl L.M., Phan H.P., Zafra-Calvo N. (2021). The role of Indigenous Peoples and local communities in effective and equitable conservation. Ecol. Soc..

[41] Parks L., Tsioumani E. (2023). Transforming biodiversity governance? Indigenous Peoples’ contributions to the convention on biological diversity. Biol. Conserv..

[42] Subramanian S., Kelemen E., De Vos A., Krause T., Mayhew M., Mead A., Nuesiri E., Perritt J., Islar M., Amaruzaman S. (2025). Inclusion in body and mind: Ensuring full participation of Indigenous Peoples and local communities in decisions related to nature. Ecol. Soc..

[43] Demetrio R.A., Cárdenas León D., Delgado C., Correa R., Espinoza R.V. (2025). Traditional ecological knowledge on stingless bees in two Ashaninka communities in the central rainforest of Peru. Ethnobiol. Conserv..

[44] Kagnew B., Assefa A., Mulugeta M., Degu A., Abebe W., Demissew S. (2025). Assessing Indigenous knowledge on diversity, socio-economy, and on-farm management practices of different yam landraces (Dioscorea cayenensis-rotundata complex) for sustainable production in Southern Ethiopia. Ethnobiol. Conserv..

[45] Moola F., Jolly H., Borah J., Roth R. (2024). The potential for Indigenous-led conservation in urbanized landscapes in Canada. Front. Hum. Dyn..

[46] Salvador D.J., Ibanez J.C. (2006). Ecology and conservation of Philippine eagles. Ornithol. Sci..

[47] Ibañez J.C., Austin B., Garnett S.T. (2016). Planning Sustainable Development within Ancestral Domains: Indigenous People’s Perceptions in the Philippines. Indigenous People and Economic Development.

[48] Jolly H., Stronza A. (2025). Insights on human−wildlife coexistence from social science and Indigenous and traditional knowledge. Conserv. Biol..

[49] (1997). Republic Act No. 8371. Official Gazette of the Republic of the Philippines. https://www.officialgazette.gov.ph/1997/10/29/republic-act-no-8371/.

[50] Camacho L.D., Gevaña D.T., Carandang A.P., Camacho S.C. (2015). Indigenous knowledge and practices for the sustainable management of Ifugao forests in Cordillera, Philippines. Int. J. Biodivers. Sci. Ecosyst. Serv. Manag..

[51] Ongpin R.F., Timones E.M. (2017). A Legal Assessment of the Protection of Indigenous Knowledge Against Biopiracy in the Philippines and ASEAN. https://www.dlsu.edu.ph/wp-content/uploads/pdf/conferences/research-congress-proceedings/2017/SEP/SEP-II-014.pdf.

[52] Maraña E.C., Arpon R.A., Capuchino I.L., Casiño R.A., Casuga K.R., Aguilar J.G. (2023). Strengthening of best practices in the preservation of cultural diversities: A phenomenological research. GSC Adv. Res. Rev..

[53] Jonsson F.A. (2014). The origins of Cornucopianism: A preliminary genealogy. Crit. Hist. Stud..

[54] Dalagan R., Hadia C.N., Bayacag J. (2000). Bayok of the Mandaya as a tool for disseminating messages on El Niño phenomenon. Davao Res. J..

[55] Camacho L.D., Combalicer M.S., Yeo-Chang Y., Combalicer E.A., Carandang A.P., Camacho S.C., De Luna C.C., Rebugio L.L. (2012). Traditional forest conservation knowledge/technologies in the Cordillera, northern Philippines. For. Policy Econ..

[56] Panopio J.K., Pajaro M., Grande J.M., Torre M.D., Raquino M., Watts P. (2021). Conservation letter: Deforestation—The Philippine eagle as a case study in developing local management partnerships with Indigenous Peoples. J. Raptor. Res..

[57] Hare D., Daniels M., Blossey B. (2021). Public perceptions of deer management in Scotland. Front. Conserv. Sci..

[58] Ma H., Zhang D., Xiao L., Wang Y., Zhang L., Thompson C., Chen J., Dowell S.D., Axmacher J.C., Lü Z. (2022). Integrating biodiversity conservation and local community perspectives in China through human dimensions research. People Nat..

[59] Cachipuendo C., Pilataxi S. (2025). The Chakana: A symbol of the Andean worldview in community water management, and a form of governance of life. Ecol. Soc..

[60] Limbaro G.R., Villareal J.F., Poclis-Villareal C.E., Santillan R.D., de Vera P.J. (2024). Understanding attitudes to urban tree conservation in Mindanao Island, Philippines. Arboric. J..

[61] Villegas J.P., Clarido A.P., Enobio V.D., Lumpapac J.D., Ibañez J.C. (2021). Local perception, values, and conservation attitude towards Brahminy kites (*Haliastur indus* Boddaert, 1783) in Tugbok district, Davao City, Philippines. Asian J. Biodivers..

[62] Catibog-Sinha C. (1986). The food preference of the Philippine deer (*Cervus philippinus* Smith, 1987) in captivity. Sylvatrop.

[63] Catibog-Sinha C. (1989). Female reproduction and parturition in penned Philippine deer (*Cervus unicolor philippinus* Smith). Philipp. J. Sci..

[64] Catibog-Sinha C. (1993). How to raise Philippine deer in captivity. A Monograph on the Philippine Deer: Debt-for-Nature Swap Project.

[65] Mattioli S., Wilson D.E., Mittermeier R.A. (2011). Family Cervidae (Deer). Handbook of the Mammals of the World.

[66] Heaney L.R., Balete D.S., Rickart E.A. (2016). The Mammals of Luzon Island: Biogeography and Natural History of a Philippine Fauna.

[67] Smith-Jones C. (2023). A Guide to the Deer of the World.

[68] Melletti M., Focardi S. (2025). Deer of the World: Ecology, Conservation and Management.

[69] Verschueren S., Bauer H., Cristescu B., Leirs H., Torres-Uribe C., Marker L. (2024). From popularity to preservation: Large carnivore potential for ecosystem conservation. Mammal Rev..

[70] Edge Davao (2011). Davao Region’s First Wildlife Rescue Center to Rise Soon. https://edgedavao.net/suburbia/2011/07/davao-regions-first-wildlife-rescue-center-to-rise-soon/.

[71] Arnett E.B., Southwick R. (2015). Economic and social benefits of hunting in North America. Int. J. Environ. Stud..

[72] Matejević M., Marković V., Kalábová M., Ristić Z., Kovačević M., Ponjiger I., Popović I. (2023). Economic impact of Roe deer tourist hunts in Vojvodina (Serbia). Cent. Eur. For. J..

[73] International Society of Ethnobiology (2006). ISE Code of Ethics (with 2008 Additions). http://ethnobiology.net/code-of-ethics/.

